# Recent survival trends in the most fatal cancers in the Nordic countries: gains in some but not in all

**DOI:** 10.2340/1651-226X.2026.45868

**Published:** 2026-06-29

**Authors:** Kari Hemminki, Frantisek Zitricky, Asta Försti, Akseli Hemminki

**Affiliations:** a Biomedical Center, Faculty of Medicine and Biomedical Center in Pilsen, Charles University in Prague, Pilsen, Czech Republic; b Division of Cancer Epidemiology, German Cancer Research Center (DKFZ), Heidelberg, Germany; c Hopp Children’s Cancer Center (KiTZ), Heidelberg, Germany; d Division of Pediatric Neurooncology, German Cancer Research Center (DKFZ), German Cancer Consortium (DKTK), Heidelberg, Germany; e Cancer Gene Therapy Group, Translational Immunology Research Program, University of Helsinki, Helsinki, Finland; f Comprehensive Cancer Center, Helsinki University Hospital, Helsinki, Finland

**Keywords:** Prognosis, fatal cancer, relative survival, incidence, sex-specific

## Introduction

Survival in cancer has improved in the developed countries. The American Cancer Society published celebrated news in January 2026: ‘Milestone 70 Percent 5-Year Survival Rate for all Cancers Combined; Largest Gains for Advanced and Fatal Cancers’ (https://pressroom.cancer.org/cancer-statistics-report-2026) [1]. Similar positive news have also been announced from the UK (Cancer in the UK: Overview 2024) [2, 3]. The Nordic countries with high-level national cancer registries also witnessed a positive development of cancer survival [4, 5]. However, there were eight solid cancers with low overall survival, and the survival rate is identical for men and women at both the 1- and 5-year marks for cancers of the hypopharynx, esophagus, stomach, liver, gallbladder, pancreas, lung, and pleura, each with a 5-year overall survival ranging between 15 and 30% [4]. The published data were collected over a 50-year period up to the year 2021 from the NORDCAN database (https://nordcan.iarc.fr/en). The eight listed cancers heavily downweight the overall survival statistics, and the 70% survival figure cited earlier can hardly be improved if survival in the common fatal cancers cannot be improved. Diverse international studies have also reported miserable survival in these eight cancers [6–9].

The current NORDCAN database extends survival data up to 2023, and we wanted to test 1- and 5-year relative survival changes in the eight fatal solid cancers in the last two 5-year periods based on the national cancer data from Denmark (DK), Finland (FI), Norway (NO), and Sweden (SE) [10]. Comparison of survival improvements within 1 and 5 years is informative of diagnosis at advanced stage disease, which usually results in early death (within the first year) [11, 12]. In 2020, the populations in the four countries were 5.8 million in DK, 5.5 million in FI, 5.4 million in NO, and 10.4 million in SE.

Before analyzing the results, we may consider how much improvement in survival can be expected per 5 years for fatal cancers. NORDCAN records survival trends since 1974, and the mean 5-year survival improvements in the four Nordic countries until 2013 were 1% for pancreatic cancer, over 1% for female lung cancer and less than 1% for male lung cancer, and close to 2% for stomach cancer.

## Methods

We used the NORDCAN database (data version: 9.5 - 06.2025), which is currently available at the IARC website (https://nordcan.iarc.fr/en/database#bloc2), a compilation of data from the Nordic cancer registries [13, 14]. We included cancers of poor survival, including esophagus, stomach, liver, gallbladder, pancreas, and lung; data for hypopharynx and pleura cancers were included as available in the database [4]; for comparison, we included all cancers (including the above ones) without the non-melanoma skin cancer (called ‘all cancer’). Relative survival data (1- and 5-year) were obtained from the database in two 5-year periods (2014–2018 and 2019–2023). Details of the survival methods are available at the NORDCAN website.

A difference in survival was considered significant when the 95% confidence intervals (CIs) were non-overlapping. The indicated changes were not significant unless specifically stated.

US data were obtained from the National Cancer Institute SEER database for White population, covering the last available period of 2015–2021 at https://seer.cancer.gov/statistics-network/explorer/application.html?site=1&data_type=4&graph_type=5&compareBy=site&chk_site_17=17&chk_site_38=38&chk_site_35=35&chk_site_47=47&chk_site_40=40&chk_site_18=18&series=9&sex=2&race=2&age_range=1&hdn_stage=101&advopt_precision=1&advopt_show_ci=on#resultsRegion1.

## Results

The number of cancer patients in DK, FI, NO, and SE for the period of 2019–2023 is shown in Supplementary Table 1. Male case numbers dominated for most of the included cancers, except for gallbladder cancer, which showed female dominance; for pancreatic and lung cancers, the case numbers were close to equal.

Relative 1- and 5-year survival rates for women are shown in Supplementary Table 2, covering all cancers combined and the eight selected cancers over the years 2014–2018 and 2019–2023. The 1-year survival rate for all cancers (without non-melanoma skin cancer) was over 80% in all countries with the best survival rate of 86.8% for SE in 2019–2023; the 5-year survival was over 70% with a best survival of 74.7% for NO. In 2019–2023, the male 5-year survival rate for all cancers in DK and FI was about 3% units lower than female survival; however, in NO and SE, the scenario was reversed, where male had an advantage (ca. 2% units), Supplementary Table 3.

**Figure 1:**
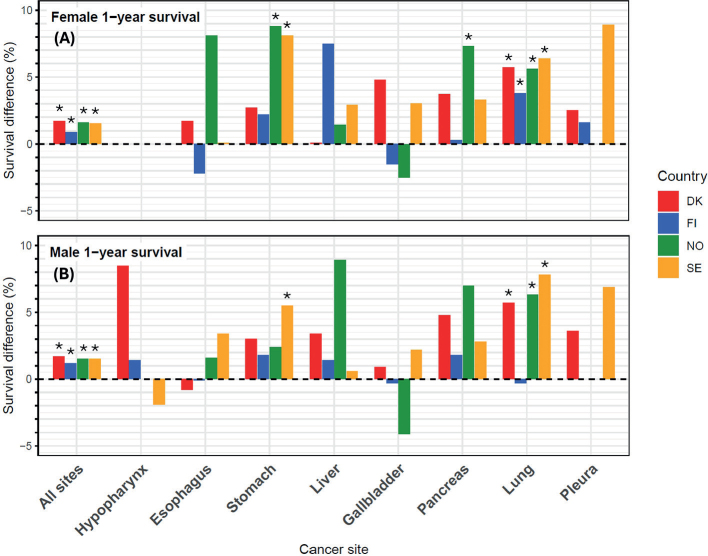
Difference in 1-year survival (in % units) in Nordic countries between 2014–18 and 2019–2023 in women (A) and in men (B). Stars on top indicate significant increases (non-overlapping CIs).

**Figure 2:**
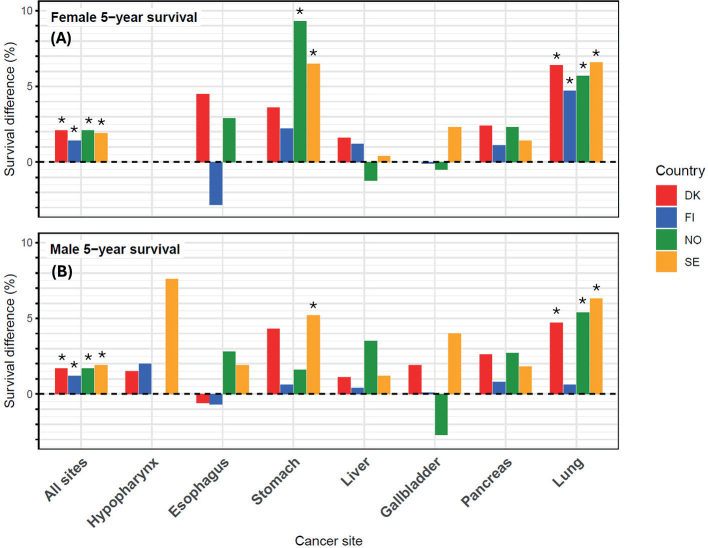
Difference in 5-year survival (in % units) in Nordic countries between 2014–18 and 2019–2023 in women (A) and in men (B). Stars on top indicate significant increases (non-overlapping CIs).

Survival differences between 2014–2018 and 2019–2023 were calculated from Supplementary Tables 2 and 3 and illustrated in Figures 1 and 2; stars on top of the bars for survival differences indicate significant changes. The improvements in 1- and 5-year survival rate for all cancers were significant in all countries (Figures 1 and 2). For all female cancers, 1-year survival improvement for women between the periods 2014–2018 and 2019–2023 was 1.7, 0.9, 1.6, and 1.5% units for DK, FI, NO, and SE, respectively; these can be compared to 5-year survival in all cancers: 2.1, 1.4, 2.1, and 1.9% units. For all male cancers, the differences between 2014–2018 and 2019–2023 for the 1-year survival rate were 1.7, 1.2, 1.5, and 1.5% units, and for 5-year survival rate, they were 1.7, 1.2, 1.7, and 1.9% units, respectively.

For all female cancers, lung cancer showed the most improvement across all Nordic countries. Specifically, 1-year survival rate increased by 5.7, 3.8, 5.6, and 6.4% units, while 5-year survival rate increase by 6.4, 4.7, 5.7, and 6.6% units for women in DK, FI, NO, and SE, respectively (Figure 1). The survival rate for stomach cancer increased in NO and SE, with 8.8 and 8.1% units in 1-year survival and 9.3 and 6.5% units in 5-year survival, respectively. The 1-year survival rate for pancreatic cancer increased by 7.3% units in NO women, but the 5-year survival rate of 2.3% units was no longer significant. A note of caution for pancreatic cancer is the increasing number of included benign neuroendocrine tumors [15].

For survival improvements in male cancers, lung cancer also showed significant progress because the survival rate improved significantly in all countries but FI (Figure 2). 1- and 5-year survival improvements for men in DK, NO, and SE were 5.7 and 4.7% units, 6.3 and 5.4% units, and 7.8 and 6.3% units, respectively. The only other male cancer for which 1- and 5-year survival improved significantly was SE stomach cancer with gains of 5.5 and 5.2% units, respectively. Additional significant male survival increases occurred only for the 1-year survival, which observed in NO for liver cancer (8.9% units) and pancreatic cancer (7.0% units) and DK for pancreatic cancer (4.8% units).

In the aforementioned results, we presented significant survival improvements, whereas Figures 1 and 2 show survival declines, and none of which were significant. These included female esophageal cancer in FI and gallbladder cancer in NO.

### Survival differences between the Nordic countries (Supplementary Tables 2 and 3)

In female cancers, the 1-year survival rate was attained best by NO for esophageal and gallbladder cancers based on non-overlapping 95% CIs. For all male cancers, SE achieved the best. NO men achieved the best 5-year survival for esophageal cancer. FI scored several worst survivals against other countries. For women, significant decreases were observed for 1-year survival in all cancers combined and lung cancers, and for 5-year survival in liver and lung cancers. For FI men, worst 1- and 5-year survival rates were noted for all cancers combined and lung cancers; in addition, they worst experienced in 1-year survival for pancreatic cancer and 5-year survival for esophageal cancer. With these negative scores, FI was however able to claim the best 5-year survival for male and female stomach cancers (overlapping 95% CIs).

### Comparison with the US SEER data

The last available US 5-year relative survival data were for the period 2015–2021. We compared the present Nordic survival data of 2019–2023 with the survival data in US White population. Almost all US survival data fell between the Nordic figures. For women, the exceptions were the US survival rate for stomach cancer (42.1%), which was better than any Nordic data. Conversely, in US female, the survival rate for pancreatic cancer (13.3%) and all cancers combined (71.8%) was below the Nordic figures. US male data for individual sites fell between the Nordic data; however, for all cancers, the US data (68.6%) were below any of the Nordic countries.

## Discussion

In the recent history, diagnosis of any of these eight cancers was a death sentence, and they continuously pose critical oncological and health policy challenges even today. Following survival in two 5-year periods in the Nordic countries, we showed a great improvement for lung cancer, which was well above improvements in all cancers. Also, NO and SE demonstrated significant survival improvements in female stomach cancer, and SE for males. Non-significant survival improvements were additionally observed for nearly all cancers (as discussed in detail in Supplementary Appendix). Among the Nordic countries, NO and SE presented the best survival, and FI reported the worst; particularly, FI ranked lowest in all lung cancer comparisons. Overall, the present survival data for the Nordic fatal cancers were at the level of the US SEER data.

## Conclusions

Survival in lung cancer increased in all countries compared with the improvements for all cancers. Survival increased also significantly for stomach and pancreatic cancers in some countries, and some improvements were seen for other cancers. For all these cancers, novel diagnostic methods and treatment procedures are urgently needed, and as these cancers are hard-to-treat, the fight should be complemented with ingenious prevention programs targeting the origins of these cancers.

## Supplementary Material





## Data Availability

The original data were obtained from the NORDCAN database (data version: 9.5 - 06.2025), which are freely available at the IARC website (https://nordcan.iarc.fr/en/database#bloc2).

## Key points

- Cancers of the esophagus, stomach, liver, gallbladder, pancreas, and lung are most fatal solid cancers.
- We analyzed survivals in these cancers between 2014 and 2025 in four Nordic countries.
- Survival in lung cancer increased in all countries compared with improvements for all cancers.
- Survival increased also for stomach and pancreatic cancers in some countries.
- The results document some success in fight against the most fatal cancers.
