# Supplementary material for: Recent survival trends in the most fatal cancers in the Nordic countries: gains in some but not in all
Source: Acta Oncol. 2026 Jun 29;65:45868. doi: 10.2340/1651-226X.2026.45868 (PMC13324887; doi:10.2340/1651-226X.2026.45868)
Supplement: Supplementary file 1 [file AO-65-45868-s1.pdf]

Supplementary material has been published as submitted. It has not been copyedited, or typeset by Acta Oncologica

SUPPLEMENTARY TABLE 1. CASE NUMBERS OF 8 CANCERS IN THE NORDIC COUNTRIES 2019-2023

	CANCER							
COUNTRY	Hypopharynx	Esophagus	Stomach	Liver	Gallbladder	Pancreas	Lung	Pleura
WOMEN								
Denmark	114	737	1213	832	786	2711	13333	114
Finland	38	516	1307	877	936	3400	6031	118
Norway	25	462	930	723	569	2316	8455	60
Sweden	66	778	1792	1425	1511	4157	12323	104
MEN								
Denmark	460	1865	2749	1979	620	3028	12420	589
Finland	170	1391	1995	2053	851	3436	8895	380
Norway	142	1304	1434	1257	514	2501	8737	338
Sweden	205	1907	2815	2978	1234	4229	10027	433

SUPPLEMENTARY TABLE 2. Female 1- and 5-year survival in the Nordic countries in 2014-2018 and 2019-2023 (NORDCAN)

	DENMARK		FINLAND		NORWAY		SWEDEN	
Site	2014-2018	2019-2023	2014-2018	2019-2023	2014-2018	2019-2023	2014-2018	2019-2023
	1-year survival							
All cancer	83.7[83.5-84.0]	85.4[85.1-85.6]	83.5[83.2-83.8]	84.4[84.2-84.7]	85.0[84.7-85.3]	86.6[86.3-86.9]	85.3[85.0-85.5]	86.8[86.6-87.0]
Esophagus	48.6[44.4-53.3]	50.3[45.6-55.4]	45.3[39.9-51.4]	43.1[38.1-48.7]	52.7[47.3-58.7]	60.8[55.5-66.6]	48.9[44.7-53.4]	49.0[44.5-54.0]
Stomach	60.6[57.6-63.7]	63.3[60.4-66.3]	61.6[58.9-64.4]	63.8[61.1-66.6]	56.4[52.9-60.2]	65.2[62.0-68.6]	55.6[53.0-58.4]	63.7[61.3-66.2]
Liver	43.6[39.8-47.7]	43.7[40.1-47.7]	40.0[36.3-44.1]	47.5[43.7-51.7]	49.2[44.5-54.4]	50.6[46.5-55.0]	50.8[47.9-53.9]	53.7[50.9-56.7]
Gallbladder	45.3[40.7-50.4]	50.1[46.1-54.5]	45.9[41.9-50.3]	44.4[40.5-48.7]	64.1[60.0-68.5]	61.6[57.4-66.2]	49.2[46.2-52.5]	52.2[49.3-55.4]
Pancreas	39.3[37.2-41.6]	43.0[40.9-45.2]	39.1[37.1-41.2]	39.4[37.5-41.4]	38.5[36.1-41.1]	45.8[43.5-48.3]	40.9[39.2-42.8]	44.2[42.5-46.0]
Lung	57.5[56.4-58.5]	63.2[62.2-64.3]	51.3[49.6-53.0]	55.1[53.5-56.7]	58.0[56.8-59.4]	63.6[62.3-64.8]	58.6[57.5-59.7]	65.0[64.0-66.1]
Pleura	62.3[53.0-73.2]	64.8[54.8-76.6]	49.0[39.1-61.5]	50.6[40.3-63.5]	-	-	50.2[39.7-63.6]	59.1[49.1-71.2]
	5-year survival							
All cancer	71.0[70.6-71.4]	73.1[72.7-73.5]	71.3[70.9-71.7]	72.7[72.3-73.1]	72.6[72.1-73.0]	74.7[74.3-75.1]	72.6[72.2-72.9]	74.5[74.2-74.8]
Esophagus	18.2[14.9-22.2]	22.7[18.2-28.2]	25.0[20.0-31.1]	22.2[17.7-27.8]	29.3[24.2-35.5]	32.2[26.6-38.8]	21.6[18.0-26.1]	21.6[17.8-26.3]
Stomach	35.7[32.6-39.1]	39.3[36.1-42.7]	39.3[36.4-42.3]	41.5[38.6-44.6]	31.7[28.3-35.6]	41.0[37.2-45.2]	30.7[28.2-33.5]	37.2[34.5-40.1]
Liver	18.1[15.1-21.8]	19.7[16.6-23.4]	11.3[8.8-14.4]	12.5[9.8-16.0]	26.0[21.9-31.0]	24.8[21.1-29.2]	24.6[22.0-27.5]	25.0[22.4-28.0]
Gallbladder	-	-	18.1[14.7-22.3]	18.0[14.8-22.1]	25.4[21.4-30.2]	24.9[21.0-29.6]	18.3[15.8-21.2]	20.6[18.0-23.6]
Pancreas	12.8[11.2-14.7]	15.2[13.4-17.3]	12.7[11.2-14.5]	13.8[12.2-15.5]	17.0[15.0-19.4]	19.3[17.2-21.7]	15.4[14.0-16.9]	16.8[15.3-18.4]
Lung	28.9[27.9-29.9]	35.3[34.2-36.5]	24.4[22.9-26.1]	29.1[27.4-30.9]	31.7[30.4-33.1]	37.4[36.0-38.8]	30.5[29.4-31.6]	37.1[35.9-38.3]

SUPPLEMENTARY TABLE 3. Male 1- and 5-year survival in the Nordic countries in 2014-2018 and 2019-2023 (NORDCAN)

	DENMARK		FINLAND		NORWAY		SWEDEN	
Site	2014-2018	2019-2023	2014-2018	2019-2023	2014-2018	2019-2023	2014-2018	2019-2023
	1-year survival							
All cancer	82.3[82.1-82.6]	84.0[83.8-84.3]	80.7[80.4-81.0]	81.9[81.6-82.2]	86.0[85.7-86.3]	87.5[87.2-87.8]	86.9[86.7-87.1]	88.4[88.2-88.6]
Hypopharynx	60.7[55.5-66.4]	69.2[64.7-74.0]	56.8[48.7-66.3]	58.2[50.3-67.3]	-	-	61.0[52.9-70.3]	59.1[51.8-67.6]
Esophagus	48.0[45.5-50.7]	47.2[44.4-50.2]	42.5[39.5-45.8]	42.4[39.5-45.5]	54.3[51.3-57.5]	55.9[53.0-58.9]	47.5[44.9-50.1]	50.9[48.1-53.9]
Stomach	57.8[55.6-60.1]	60.8[58.8-62.9]	56.4[54.0-58.9]	58.2[55.9-60.7]	59.0[56.3-61.8]	61.4[58.6-64.3]	56.2[54.2-58.3]	61.7[59.7-63.7]
Liver	43.9[41.1-46.8]	47.3[44.8-50.0]	42.4[39.6-45.3]	43.8[41.3-46.4]	45.0[41.5-48.9]	53.9[50.7-57.3]	53.6[51.6-55.6]	54.2[52.2-56.3]
Gallbladder	51.0[46.5-55.8]	51.9[47.7-56.4]	45.5[41.5-49.9]	45.2[41.3-49.4]	65.9[61.8-70.4]	61.8[57.2-66.9]	56.4[53.3-59.7]	58.6[55.7-61.6]
Pancreas	35.6[33.6-37.7]	40.4[38.5-42.4]	32.8[30.9-34.7]	34.6[32.7-36.6]	36.1[33.7-38.6]	43.1[40.9-45.5]	39.1[37.3-40.9]	41.9[40.2-43.6]
Lung	49.1[47.9-50.2]	54.8[53.6-56.0]	43.3[42.0-44.7]	43.0[41.6-44.6]	50.5[49.1-51.9]	56.8[55.5-58.2]	51.0[49.8-52.2]	58.8[57.6-60.1]
Pleura	59.9[54.8-65.4]	63.5[56.3-71.7]	-	-	-	-	51.2[44.6-58.6]	58.1[51.0-66.3]
	5-year survival							
All cancer	68.8[68.4-69.2]	70.5[70.1-70.9]	67.9[67.5-68.4]	69.1[68.6-69.5]	74.5[74.1-75.0]	76.2[75.8-76.7]	75.4[75.1-75.8]	77.3[77.0-77.6]
Hypopharynx	33.2[27.8-39.6]	34.7[28.2-42.8]	34.4[25.5-46.3]	36.4[27.5-48.2]	-	-	22.3[15.3-32.4]	29.9[22.3-40.0]
Esophagus	20.4[18.2-22.8]	19.8[17.5-22.5]	14.7[12.5-17.3]	14.0[11.8-16.5]	23.4[20.7-26.4]	26.2[23.4-29.3]	16.7[14.5-19.2]	18.6[16.4-21.2]
Stomach	27.9[25.7-30.2]	32.2[30.0-34.6]	32.7[30.3-35.3]	33.3[30.8-35.9]	30.4[27.7-33.3]	32.0[29.2-35.2]	27.0[25.0-29.1]	32.2[30.1-34.4]
Liver	16.3[14.1-18.9]	17.4[15.2-19.8]	13.4[11.4-15.8]	13.8[11.9-16.0]	20.4[17.5-23.9]	23.9[20.7-27.4]	22.9[21.2-24.8]	24.1[22.3-26.1]
Gallbladder	18.9[15.3-23.4]	20.8[17.0-25.5]	14.5[11.4-18.4]	14.6[11.6-18.3]	26.8[22.7-31.7]	24.1[20.0-28.9]	22.3[19.5-25.4]	26.3[23.3-29.6]
Pancreas	11.2[9.8-12.9]	13.8[12.2-15.6]	10.5[9.0-12.1]	11.3[9.9-13.0]	15.6[13.6-17.8]	18.3[16.4-20.6]	14.7[13.3-16.3]	16.5[15.1-18.1]
Lung	22.3[21.2-23.4]	27.0[25.8-28.2]	16.6[15.4-17.8]	17.2[16.0-18.5]	24.4[23.2-25.8]	29.8[28.4-31.2]	22.9[21.8-24.2]	29.2[27.9-30.6]
