# Supplementary material for: Recent survival trends in the most fatal cancers in the Nordic countries: gains in some but not in all
Source: Acta Oncol. 2026 Jun 29;65:45868. doi: 10.2340/1651-226X.2026.45868 (PMC13324887; doi:10.2340/1651-226X.2026.45868)
Supplement: Supplementary file 2 [file AO-65-45868-s2.pdf]

Supplementary material has been published as submitted. It has not been copyedited, or typeset by Acta Oncologica

SUPPLEMENTARY DISCUSSION

The recent US study on cancer statistics identified 5 cancers with the lowest 5-year survival but for which survival improvement had been higher than for the other cancers in the past 40 years (1). The cancers were those of the esophagus, liver, lung, pancreas and stomach, which were also among the most fatal solid cancers in the Nordic countries (2). In addition to the above cancers, the Nordic list included rare gallbladder, hypopharyngeal and pleural cancers. Survival in cancer is critically dependent on the stage at diagnosis which data however are not available in NORDCAN (3, 4). In the Netherlands Cancer Registry 1-year survival in de novo metastatic disease (metastasis at diagnosis) has increased somewhat even in fatal cancers but hardly at all for 5-year survival (5). In the US SEER database, lung and stomach cancer 5-year survival is about 70% in localized cancers but only 10% when diagnosed at a metastatic stage (1).

Although we lack stage data, comparison of 1- and 5-year survival figures may be informative of stage; early deaths (low 1-year survival) are often due to high-stage cancers. Among the present 8 cancers, 1-year survival in 2019-2023 varied between 40 and 60%, which could suggest that half of the patients had been diagnosed at an advance stage and died of the cancer or the related complications. We similarly recorded 5-year survivals between 15 and 35%, implying that approximately half or those who survived the first year died during years 2 to 5, probably due to relapse or late metastases. Survival probabilities depended on sex and cancer, female 1-year survival for stomach and lung cancers was over 60% compared to 5-year survival of 40% (stomach) and 35% (lung). In males 5-year survival was barely over 30% for stomach cancer and under 30% for lung cancer. For the most fatal cancers of the pancreas and liver, male DK and FI 5-year survival was some 15%. Among the significant survival improvements listed in **Supplementary Tables 2 and 3**, lung cancer was unique because of increases for 1- and 5-year survival in almost every comparison. However, considering other cancers, 7 country-specific significant survival improvements were noted for 1-year survival and 3 for 5-year survival, mainly in NO and SE, suggesting that early detection and well working patient care pathways are contributing.

The positive developments in lung cancer in general and female excess in particular have probably many explanations at the time when no screening has been organized in the Nordic countries (6). Lung cancer histology has changed towards the therapeutically amenable adenocarcinoma which in SE women has increased to 60%, compared to men of 54% and the proportion of patients diagnosed with metastases has decreased to some 50% [20230921_nlcr_nationell_rapport2022.pdf (cancercentrum.se)]. Thorax-CT is increasingly applied and this has increased detection of early A1 lung cancers (7). Therapeutic developments also extend to other histologies for which immunotherapy and chemoimmunotherapy are being used. Previous clinical and survival studies on lung cancer from the Nordic countries describe periodic improvements in diagnostics and treatment of lung cancer (8-11). These report that continuous focus has been devoted to facilitation of care pathways and to removal of small treatment units in favor of centralized treatment centers operated by multidisciplinary teams. An international study on lung cancer covering also UK, Canadian and Australian cancer registries in years 2012-2015 reported that time interval from

first symptoms to initiation of curative treatment was shortest in DK and NO, and the interval from first medical contacts to definite diagnosis was shortest in SE (12).

Sensitive CT and PET have become parts of standard diagnostics, complemented with molecular analysis of histological samples for therapeutically relevant mutations in genes, such as epidermal growth factor receptor (EGFR) and fusion products in genes such as anaplastic lymphoma kinase (ALK). Minimal invasive surgery is applied in early-stage lung cancer, in stages II-III a wide armamentarium of radio- (stereotactic radiotherapy), chemo- and immunotherapy are used (13). In stage IV, depending on the condition of the patient, the options include surgery, chemotherapy (e.g., platinum), targeted therapy or immunotherapy (indicated for PDL1 positive patients or if tumor mutation burden is high) (13-16). In general, the Nordic countries try to follow the ESMO guidelines for lung cancer (15, 16). The strong development of 1-year survival for lung cancers of 5 % units, documented in **Fig. 1**, and reaching over 60% for women and over 50% for men is the key message of this study.

Another cancer with success in NO and SE females and SE males was stomach cancer, and also in NO, esophageal cancer. NO has also excelled in a recent survival study on these cancers (17). Current treatment options for stomach cancer include surgical resection with chemotherapy and radiotherapy in localized disease, or in Her2 positive disease targeted therapy; in metastatic cancer chemotherapy may be used (18, 19). Recent options are anti-angiogenic therapy and immunotherapy (19). Treatment of stomach and esophageal cancers has been increasingly centralized to specialist clinics with advanced endoscopic and other surgical techniques which are shared by these cancers (18, 20). A recent NO study reported that curative treatment was offered to 60% of stomach and 50% of esophageal cancer patients (21). The decisions on selection between curative or palliative treatment depended on the presence of distant metastases. According to a SE report, the trends in treatment of these cancers have involved an increase in minimally invasive surgery, decrease in resection rates (excluding endoscopic resections) and increase in application of preoperative treatment with chemotherapy for stomach cancer and with chemo-radiotherapy for esophageal cancer (22). A further positive feature for survival in stomach and esophageal cancers is that improvements have been achieved even among elderly patients particularly after 2010 (17).

We have focused on survival improvements but in **Fig. 1** and **2** there are also some non-significant negative results, for FI women in esophageal cancer and for NO men in gallbladder cancer.

The Nordic countries have a relatively large immigrant population which is highest in SE (23). Most immigrant populations which entered SE had lower cancer incidence compared to the natives but incidence and survival rates depend on the origin of immigrants (23). It is however unlikely that any single immigrant group would significantly influence the present results.

The study period included the main Covid 19 epidemic in the spring of 2020, when incident cancers decreased in all Nordic cancer registries, most in SE with least population containment (24). There was a rebound of cancer cases later in 2020 but it did not fully compensate for the earlier deficits. We assume that the small deficit did not influence the present results.

In conclusion, we analyzed survival changes between two consecutive 5-year periods up to 2023 in 8 most fatal cancers of which some were rare and lacking data. Survival improvement was significant for lung cancer in each country, more for women than for men. For females, 1- and 5-year lung cancer survival improvements were about 5 and 6 % units between the two periods, compared to all cancer of 1.5 and 2 % units, respectively. Regarding other individual sites, Norway and Sweden demonstrated significant survival improvements in stomach cancer, and Norway also in pancreatic cancer. However, non-significant survival improvements were observed for most cancers. No positive evidence was found for esophageal cancer in Finland and gallbladder cancer in Norway. More significant improvements were found for 1- than for 5-year survival. To fight these fatal cancers continuous efforts are needed in early detection, facile clinical handling and novel therapeutics. Also, for these fatal cancers primary prevention would be highly rewarding.

LITERATURE

1. Siegel RL, Kratzer TB, Wagle NS, Sung H, Jemal A. Cancer statistics, 2026. CA Cancer J Clin. 2026;76(1):e70043.
2. Hemminki J, Försti A, Hemminki A, Hemminki K. Survival trends in solid cancers in the Nordic countries through 50 years. Eur J Cancer. 2022;175:77-85.
3. McPhail S, Johnson S, Greenberg D, Peake M, Rous B. Stage at diagnosis and early mortality from cancer in England. Br J Cancer. 2015;112 Suppl 1(Suppl 1):S108-15.
4. Forjaz G, Ries L, Devasia TP, Flynn G, Ruhl J, Mariotto AB. Long-term Cancer Survival Trends by Updated Summary Stage. Cancer Epidemiol Biomarkers Prev. 2023;32(11):1508-17.
5. Luyendijk M, Visser O, Blommestein HM, de Hingh I, Hoebers FJP, Jager A, et al. Changes in survival in de novo metastatic cancer in an era of new medicines. J Natl Cancer Inst. 2023.
6. Hemminki K, Zitricky F, Forsti A, Hemminki O, Hemminki A. Age-specific survival in lung cancer in the Nordic Countries through a half century. . J Biomed Res Environ Sci. 2024;5:996-1005.
7. Borg M, Hilberg O, Andersen MB, Weinreich UM, Rasmussen TR. Increased use of computed tomography in Denmark: stage shift toward early stage lung cancer through incidental findings. Acta Oncol. 2022;61(10):1256-62.
8. Lundberg FE, Ekman S, Johansson ALV, Engholm G, Birgisson H, Ólafsdóttir EJ, et al. Trends in lung cancer survival in the Nordic countries 1990-2016: The NORDCAN survival studies. Lung Cancer. 2024;192:107826.
9. Sachs E, Sartipy U, Jackson V. Sex and Survival After Surgery for Lung Cancer: A Swedish Nationwide Cohort. Chest. 2021;159(5):2029-39.
10. Solberg S, Nilssen Y, Brustugun OT, Grimsrud TK, Haram PM, Helbekkmo N, et al. Increase in curative treatment and survival of lung cancer in Norway 2001-2016. Eur J Epidemiol. 2019;34(10):951-5.
11. Tichanek F, Försti A, Hemminki O, Hemminki A, Hemminki K. Survival in Lung Cancer in the Nordic Countries Through A Half Century. Clinical epidemiology. 2023;15:503-10.
12. Menon U, Vedsted P, Zalounina Falborg A, Jensen H, Harrison S, Reguilon I, et al. Time intervals and routes to diagnosis for lung cancer in 10 jurisdictions: cross-sectional study findings from the International Cancer Benchmarking Partnership (ICBP). BMJ Open. 2019;9(11):e025895.
13. Maconachie R, Mercer T, Navani N, McVeigh G. Lung cancer: diagnosis and management: summary of updated NICE guidance. Bmj. 2019;364:l1049.

14. Löfling L, Bahmanyar S, Kieler H, Lambe M, Wagenius G. Temporal trends in lung cancer survival: a population-based study. Acta Oncol. 2022;61(5):625-31.
15. Hendriks LE, Kerr KM, Menis J, Mok TS, Nestle U, Passaro A, et al. Non-oncogene-addicted metastatic non-small-cell lung cancer: ESMO Clinical Practice Guideline for diagnosis, treatment and follow-up. Annals of oncology : official journal of the European Society for Medical Oncology. 2023;34(4):358-76.
16. Hendriks LE, Kerr KM, Menis J, Mok TS, Nestle U, Passaro A, et al. Oncogene-addicted metastatic non-small-cell lung cancer: ESMO Clinical Practice Guideline for diagnosis, treatment and follow-up. Annals of oncology : official journal of the European Society for Medical Oncology. 2023;34(4):339-57.
17. Hemminki K, Zitricky F, Försti A, Hemminki O, Liska V, Hemminki A. Survival improvements in esophageal and gastric cancers in the Nordic countries favor younger patients. Cancer medicine. 2024;13(15):e7365.
18. Allum W, Lordick F, Alsina M, Andritsch E, Ba-Ssalamah A, Beishon M, et al. ECCO essential requirements for quality cancer care: Oesophageal and gastric cancer. Crit Rev Oncol Hematol. 2018;122:179-93.
19. Smyth EC, Nilsson M, Grabsch HI, van Grieken NC, Lordick F. Gastric cancer. Lancet. 2020;396(10251):635-48.
20. Kalff MC, Gottlieb-Vedi E, Verhoeven RHA, van Laarhoven HWM, Lagergren J, Gisbertz SS, et al. Presentation, Treatment, and Prognosis of Esophageal Carcinoma in a Nationwide Comparison of Sweden and the Netherlands. Annals of surgery. 2021;274(5):743-50.
21. Kolstad A, Emanuel G, Hjortland GO, Nilssen Y, Ulvestad M, Areffard A, et al. Long-term trends in the clinical management and outcomes of patients with gastroesophageal cancer in Norway. Acta Oncol. 2025;64:540-9.
22. Jeremiasen M, Linder G, Hedberg J, Lundell L, Björ O, Lindblad M, et al. Improvements in esophageal and gastric cancer care in Sweden-population-based results 2007-2016 from a national quality register. Dis Esophagus. 2020;33(3).
23. Mousavi SM, Hemminki K. Cancer incidence, trends, and survival among immigrants to Sweden: a population-based study. Eur J Cancer Prev. 2015;24 Suppl 1:S1-s63.
24. Johansson ALV, Larønningen S, Skovlund CW, Kristiansen MF, Mørch LS, Friis S, et al. The impact of the COVID-19 pandemic on cancer diagnosis based on pathology notifications: A comparison across the Nordic countries during 2020. Int J Cancer. 2022;151(3):381-95.
